# Anticipating the Direction of Soccer Penalty Shots Depends on the Speed and Technique of the Kick

**DOI:** 10.3390/sports6030073

**Published:** 2018-07-29

**Authors:** Andrew H. Hunter, Sean C. Murphy, Michael J. Angilletta, Robbie S. Wilson

**Affiliations:** 1 School of Biological Sciences, The University of Queensland, St Lucia, QLD 4067, Australia; r.wilson@uq.edu.au; 2 Melbourne School of Psychological Sciences, The University of Melbourne, Parkville, VIC 3052, Australia; murphy.s1@unimelb.edu.au; 3 School of Life Sciences, Arizona State University, Tempe, AZ 85281, USA; Michael.Angilletta@asu.edu

**Keywords:** soccer, anticipation, speed, trade-off, goalkeeper, penalty, shooting

## Abstract

To succeed at a sport, athletes must manage the biomechanical trade-offs that constrain their performance. Here, we investigate a previously unknown trade-off in soccer: how the speed of a kick makes the outcome more predictable to an opponent. For this analysis, we focused on penalty kicks to build on previous models of factors that influence scoring. More than 700 participants completed an online survey, watching videos of penalty shots from the perspective of a goalkeeper. Participants (ranging in soccer playing experience from never played to professional) watched 60 penalty kicks, each of which was occluded at a particular moment (−0.4 s to 0.0 s) before the kicker contacted the ball. For each kick, participants had to predict shot direction toward the goal (left or right). As expected, predictions became more accurate as time of occlusion approached ball contact. However, the effect of occlusion was more pronounced when players kicked with the side of the foot than when they kicked with the top of the foot (instep). For side-foot kicks, the direction of shots was predicted more accurately for faster kicks, especially when a large portion of the kicker’s approach was presented. Given the trade-off between kicking speed and directional predictability, a penalty kicker might benefit from kicking below their maximal speed.

## 1. Introduction

Sport scientists commonly measure maximal performances such as fastest speed, highest leap, or farthest throw, because such parameters are thought to reflect performance in a game or event. However, increases in one kind of performance may be associated with decreases in another. For example, moving faster usually reduces agility [1,2] and accuracy [3]. Throwing darts [4], kicking soccer balls [5], and pitching in baseball or cricket [6] are all subject to a trade-off between speed and accuracy. Thus, sporting success does not rely simply on maximal performance but is affected by trade-offs that can be managed to optimise overall success.

In soccer, a potential trade-off between the speed and unpredictability of an action could influence success in penalty kicks. In this situation, unpredictability is advantageous, and soccer players are more likely to score on a penalty kick if they can disguise the direction of the kick [7]. During penalties, goalkeepers use cues presented by the kicker to predict shot direction before the ball moves [8,9,10]. If a shooter kicks as fast as possible, their range of motion increases compared to a slower kick [11,12], exaggerating visual cues used by the goalkeeper and improving their accuracy in predicting the direction of the shot [13].

The ability to anticipate ball direction has been studied in a range of sports, including badminton [14], tennis [15], squash [16], and soccer [8,17,18]. In most of these studies, a subject is shown a video in which a portion of the opponent’s (shooter’s) movement has been occluded. In this way, researchers can determine whether a shooter’s movements reveal their placement of the ball. Not surprisingly, subjects predict direction more accurately when they have more visual information about a shot. Two studies manipulated movement amplitude, finding it influenced subjects’ ability to predict shot direction in soccer [13] but not tennis [15]. As these studies manipulated movement amplitude, not shot speed, the relationship between speed and unpredictability is unclear. A better understanding of this phenomenon could assist kickers in selecting (and training for) the shooting strategy that maximises their chance of scoring. While various factors contribute to the outcome of a penalty shot [13,19,20,21], understanding any variable that increases or decreases the likelihood of goalkeepers anticipating shot direction is beneficial for shooters.

In this study, we quantified the trade-off between speed and unpredictability using videos of soccer penalties. By manipulating the speed of a kick with human actors, we investigated the relationship between speed and unpredictability more rigorously than in previous studies of soccer or tennis. Controlling for footedness [20] and approach angle [20,22], soccer players were recorded from the perspective of a goalkeeper while shooting penalties at various speeds. These videos were presented to subjects in a computer-based survey in which participants guessed the direction of each kick. We were also interested in how the relationship between speed and unpredictability might be mediated by the amount of information observers receive, as shot direction is easier to predict when predictions are made closer to the shooter’s foot contacting the ball [7,13,17]. Therefore, we manipulated the endpoint of each video so that it varied from the point at which the shooter’s foot contacted the ball to −0.4 s before contact. Shooters used both kick techniques seen in soccer: side-foot and instep kicks. We predicted participants would be more likely to guess the direction of faster shots compared to slower shots, and that predictions made closer to ball contact more likely to be correct than predictions made earlier in the shooter’s kicking action.

## 2. Materials and Methods

The survey was an online-based community convenience sample constructed using Qualtrics [23] and the video hosting site Vimeo. A link to the survey was distributed via email, Facebook, Twitter, and the University of Queensland online magazine. Informed consent was obtained and the methods and protocols for this experiment were approved by the Behavioural and Social Sciences Ethical Review Committee, University of Queensland (project ID-2012001078).

Prior to completing the survey, participants were asked their age, gender, soccer playing experience before the age of 18, soccer playing experience after the age of 18, and if their soccer playing experience was predominantly as a goalkeeper or outfield player (see Appendix A).

### 2.1. Survey Task

Participants watched 60 videos of soccer players taking penalty shots. Each video was a single penalty shot filmed from the perspective of a goalkeeper. Each video commenced just prior to the start of the shooter’s run-up and ended at various points up until the shooter’s foot contacted the ball, thereby removing any information about the ball’s trajectory. After viewing each video, participants were asked to decide whether the shot went to their left or their right. Instructions were provided at the beginning of the survey (see Appendix A), followed by 10 practice videos, then 60 test videos. Participants received feedback during the 10 practice videos informing them if their answer was correct. They did not receive feedback during the test phase.

### 2.2. Video Production

**Figure 1:**
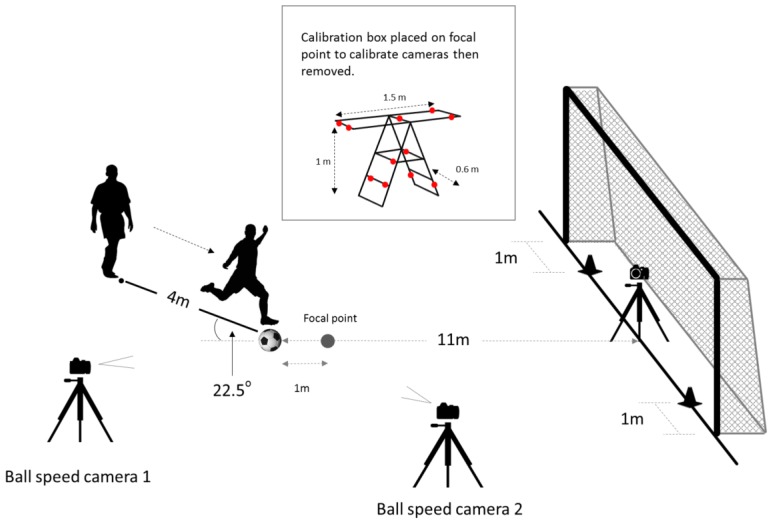
Graphical representation of experimental setup used to produce videos used in the survey.

Ten right-footed soccer players from the University of Queensland Football Club were recruited to produce the video clips watched by participants. Video footage was captured on a camera (Panasonic Lumix DMC-TZ40, 50 fps, resolution 1920 × 1080, Panasonic, Kadoma, Japan) positioned 1.5 m off the ground in the middle of a soccer goal facing the penalty spot (Figure 1). A designated starting spot for all shooters was marked on the ground 4 metres behind the penalty spot at an angle of 22.5° (Figure 1). A line was drawn on the ground between the penalty spot and this mark. Players were instructed to execute shots to both sides of the goal aiming at a marker placed 1 metre inside either goal-post. Both side-foot and instep kicks were executed across a range of shot speeds (~50–100% of an individual’s maximum kicking speed). Shooters were instructed to (1) commence their run-up from the designated starting spot and approach the ball along the drawn line, (2) not use any deception or try to conceal the direction they were shooting but concentrate on accuracy and shoot with a natural kicking motion, and (3) not look at their intended target for the period 2 s before they commenced their run-up until after they had completed their shot.

### 2.3. Video Analysis

To measure ball speed, we used the DLTcal5 and DLTdv5 packages of MATLAB [24]. First, two high-speed cameras (Panasonic Lumix DMC-TZ40) were calibrated to a three-dimensional space. Then, coordinates (x,y,z) were extracted from subsequent footage taken with the calibrated cameras. To calibrate the cameras, an ‘imaginary’ focal point was designated at 1 m in front of the penalty spot (i.e., 10 m from the goal). An 11-point calibration box (1.5 m × 1 m × 0.6 m) was centred on the focal point, thereby filling the space through which the ball travelled (Figure 1). Two high-speed cameras, each on a 1 m tripod, were oriented 90 degrees from each other and facing the focal point. The first camera was positioned approximately 3 m behind the penalty spot and 3 m to the side to avoid impeding the kickers’ approach. The second camera was placed 3 m in front of the penalty spot and 3 m to the side, perpendicular to the ball’s trajectory. After positioning and filming the calibration box with both cameras, the box was removed. Each kick was then recorded on the cameras filming at identical frame rates (100 fps). In MATLAB, the ball’s centre was extracted from six frames that spanned the first 0.06 s after the foot struck the ball. With these positional data the distance the ball travelled between each frame was first calculated. Then, knowing the frame rate, we calculated the speed of the ball between each frame. The average of these six velocities gave our measure of ball speed. For every player, the speed of each shot was converted to a percentage of their maximum speed for that side of the goal (left or right) and shooting technique (side-foot or instep).

### 2.4. Video Selection

For every player, twelve shots were selected to be in the survey—three shots for each combination of side aimed at and kicking technique (left and side-foot; right and side-foot; left and instep; right and instep). The three shots selected for each group were of varying speeds and categorised as slow, medium, or fast in order of increasing speed. The shot speeds used across all shooters for each kick technique were (Mean ± Standard Deviation): Slow side-foot, 64.9% ± 6.54%; medium side-foot, 84.8% ± 3.9%; fast side-foot, 99.5% ± 1%; slow instep, 62.4% ± 5.7%, medium instep, 83.7% ± 6%; fast instep, 99.6% ± 1.2%. The video of each shot was edited with the open-source software program Kinovea (v0.8.15, Kinovea, France). Original videos were converted to 30 frames per second to enable uploading to Vimeo. Each video was then edited to start 2 s before the shooter commenced their run-up toward the ball. The videos ended at one of 5 points in time (occlusion time): (1) At ball contact (Supplementary video S0), (2) −0.1 s before ball contact (Supplementary video S1), (3) −0.2 s before ball contact (Supplementary video S2), (4) −0.3 s before ball contact (Supplementary video S3), or (5) −0.4 s before ball contact (Supplementary video S4). During the survey, the screen went blank after each video ended and participants were asked to infer the direction of the shot.

Combining the edited videos for 10 kickers yielded a pool of 600 videos. In designing the survey, we wanted to keep the following conditions consistent among participants: (1) An even spread of shots that went left or right, (2) an even spread of side-foot and instep shots, (3) an even spread of occlusion times, (4) an even spread of shot speeds, (5) shots randomized among kickers, and (6) not more than one occlusion of each original video. With this is in mind, ten groups of 60 videos were created that satisfied these conditions with no video repeated within or across groups. Participants were randomly assigned to watch one of these video groups with videos in random order. The 10 practice trials were produced from shots separate from those included in the test phase and included shots aimed left and right across a range of speeds, kick techniques, and occlusion times.

### 2.5. Post-Survey Feedback

At the completion of the survey, participants were given feedback on the number of shots they guessed correctly. This was broken down into five ‘difficulty’ levels corresponding with the five occlusion time conditions. The average number of correct guesses for each difficulty level from a pilot sample was also presented.

### 2.6. Penalty Shootout Analysis

**Figure 2:**
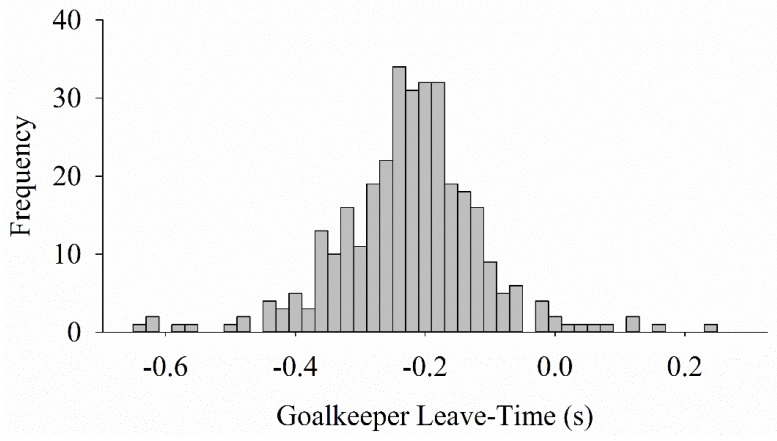
Frequency distribution of goalkeeper leave-time from 330 penalty kicks in professional/international matches. Negative values are before ball contact.

Previous studies of penalty kicks report that goalkeepers dive at different times in matches [25,26] and under experimental conditions [17,27]. However, no study presents a distribution describing the variance in time, relative to ball contact, goalkeepers choose to dive in matches. To estimate this distribution, we analysed 330 penalty shots from existing footage of 34 penalty shootouts from professional competitions (e.g., Fédération Internationale de Football Association {FIFA} World Cup, Union of European Football Associations {UEFA} Champions League, Africa Cup of Nations), with 41 countries or clubs represented in the sample. We sourced video of penalty shootouts from Youtube, and using Kinovea, two times were extracted from each penalty: (1) When the shooter’s foot contacted the ball and (2) when the goalkeeper initiated their dive to a side. Some goalkeepers make movements unrelated to their final dive direction during the shooter’s run-up (e.g., bobbing up and down, moving laterally side-to-side). These movements were ignored until the goalkeeper initiated their final dive. We then calculated the time goalkeepers first moved relative to ball contact (leave-time). The frequency distribution of leave-time is presented in Figure 2 (M = −0.22 s, SD = 0.11 s). From this distribution, the range of occlusion times we selected (−0.4 s to 0 s) represents the range of leave-times commonly used by professional goalkeepers in matches.

### 2.7. Statistical Analysis

Due to a low sample size (N = 3), participants classifying their soccer playing experience over the age of 18 as professional were removed from analysis. A one-way ANOVA and Tukey Honest Significant Difference (95% Confidence Intervals) [28] was initially used to detect significant effects of soccer playing experience over the age 18 on correctly guessing shot direction. A generalised linear model (GLM) with a binomial distribution [28] was used to relate the probability of correctly guessing a shot’s direction to its speed (fast, medium, or slow), kick technique (side-foot or instep), and occlusion time (−0.4 s, −0.3 s, −0.2 s, −0.1 s, or 0.0 s before ball contact). To estimate the most likely effect of each variable in the GLM, we used multi-model inference based on information theory [29,30]. Initially, we estimated parameters from the full model containing all main effects and interactions. Then, we estimated the parameters of every sub-model, including the null model, using the MuMIn library of R [31]. Based on the Akaike weight of each model, which gives the likelihood that a model best describes the data (Table 1), we calculated a weighted average value for each parameter among all models (Table 2). These values were then used to calculate the expected probability under each condition of the experiment. Multi-model inference estimates the effects of variables more accurately than null hypothesis testing, as all possible models (including the null) contribute to the most likely value of each parameter.

## 3. Results

**Figure 3:**
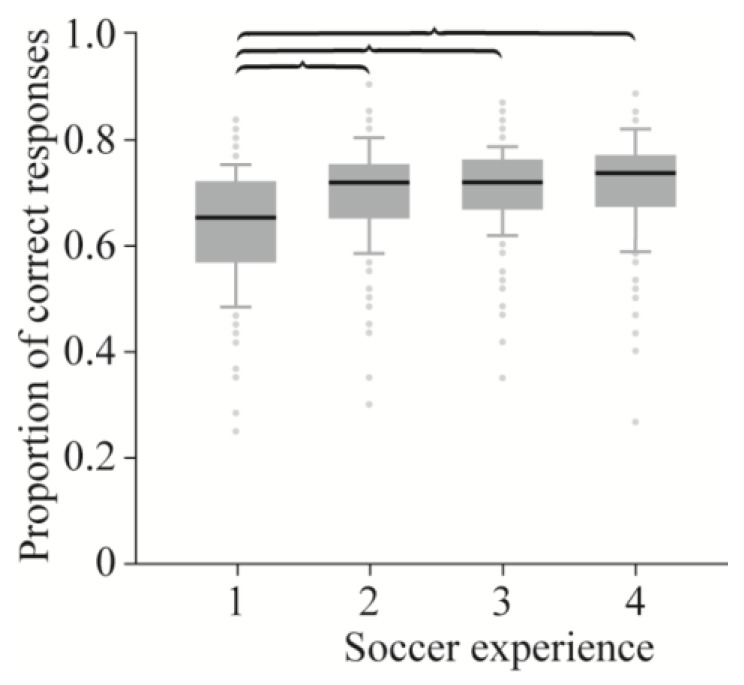
Success rates of participants grouped according to those who were over 18 years old with soccer playing experience. (1) Never played, N = 166. (2) Played socially, N = 215. (3) Amateur player, N = 213. (4) Semi-professional player, N = 100. See Appendix A for full descriptions. Plotted are the median, 10th, 25th, 75th, 90th percentile, and outliers. ANOVA revealed a significant difference among groups, F (3690) = 25.61, *p* < 0.001. Braces show significant differences among groups identified by Tukey HSD (95% CI). All significant differences are *p* < 0.001 (see Appendix Table A1 for further details).

Of the 709 participants who completed the survey, 550 were male, 155 were female, and 4 participants did not define their gender. Their ages ranged from 6 to 70 years, with 37 participants being under the age of 18. To ensure that results were relevant, we excluded participants under the age of 18 years or who had never played soccer (Figure 3), leaving 521 participants (male = 435, female = 82) for analysis. As the included participants did not differ in the proportion of correct responses based on soccer playing experience over the age of 18 (Figure 3), this variable was not included in the GLM. Seventy-seven participants reported experience as a goalkeeper after the age of 18.

**Figure 4:**
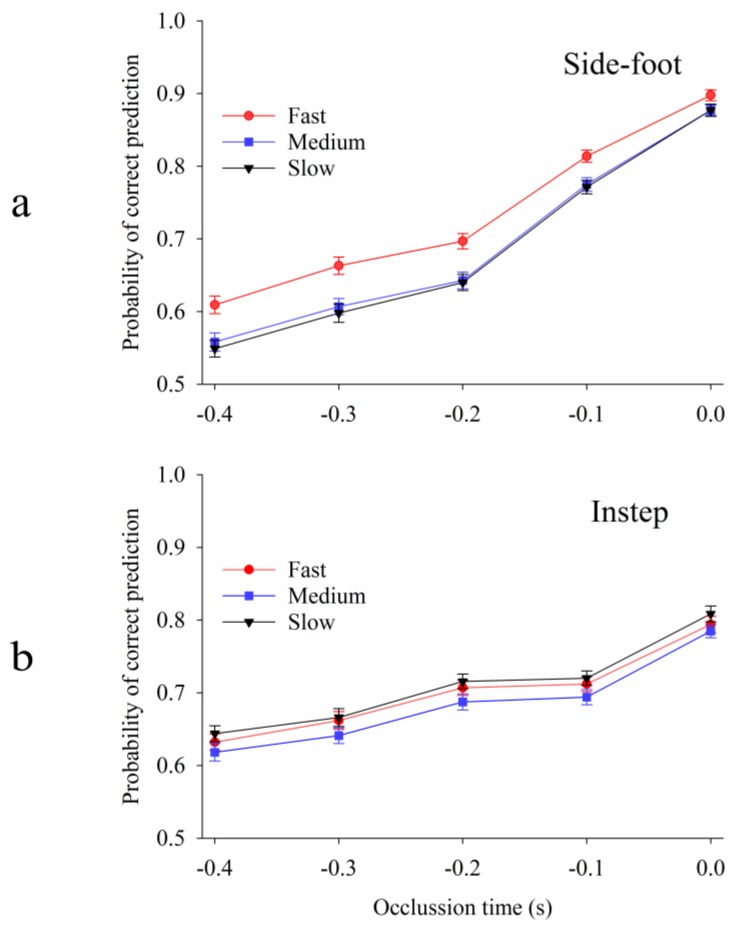
Probability of correctly guessing shot direction dependent on occlusion time and shot speed. Side-foot and instep shots are plotted separately. Probabilities and standard error bars were calculated using averaged parameter estimates from statistical model. (**a**) Side-foot shots. (**b**) Instep shots.

As expected, participants were better at predicting the direction of shots at later occlusion times (Figure 4). At −0.4 s before ball contact, participants correctly guessed shot direction 55% to 64% of the time, depending on the kick technique and speed of the shot (Figure 4). However, when shown ball contact, participants were successful ≈80% or ≈90% of the time for shots with instep or side-foot, respectively.

The effect of occlusion was greater for side-foot shots than instep shots, particularly for slow and medium-paced side-foot shots. At early occlusion times (−0.4 s, −0.3 s), participants predicted the direction of 55% to 61% of side-foot shots (slow and medium-paced), compared to 62% to 67% of instep shots (all speeds). As occlusion time approached ball contact, the predictability of side-foot shots increased at a greater rate than instep shots, with side-foot shots reaching a maximum of 90% at ball contact compared to 81% for instep shots (Figure 4).

Faster shots were easier to predict than medium and slow shots when shooters used a side-foot kicking technique. This effect was most pronounced at early occlusion times. For example, at −0.4 s before ball contact, participants correctly guessed 61% of fast shots compared to only 55% of slow shots and 56% of medium shots (Figure 4). This difference gradually reduced as occlusion time approached ball contact, at which point participants guessed 88% of slow and medium shots, compared to 90% of fast shots. We found similar patterns when only data from participants with goalkeeping experience were analysed (see Supplemental Figure S1).

## 4. Discussion

Goalkeepers face a clear trade-off between moving early and moving in the correct direction. To increase the chance of intercepting the ball, goalkeepers typically begin to move several hundred milliseconds before the ball moves [17,27]. As with previous experiments, we confirm that earlier movements reduce the ability to predict shot direction. Under all conditions, participants in our study were better at predicting shot direction when given more video footage of the kicker’s approach. The foot’s final trajectory at contact is a reliable indicator of the ball’s trajectory [32,33]. Not surprisingly, participants who viewed a shot to the point of ball contact were likely to guess its direction correctly, regardless of kicking speed. In a match, keepers who delay their movement will receive more accurate information about shot direction, improving anticipation.

We show that the likelihood of goalkeepers moving in the correct direction depends on an interaction between the keeper’s strategy (leave-time) and the shooter’s strategy (technique, speed). If goalkeepers move late, instep shots of any speed are the least predictable. If goalkeepers move early, slow/medium side-foot shots reveal less about shot direction than all other shots. Considering the average leave-time for professional goalkeepers we identified (−0.22 s), slow/medium side-foot shots are the least predictable at this time (Figure 4). Previous studies show that kicking with the side of the foot [30,34], and more slowly [5,30], yields greater accuracy. Taken together, we show that kickers may use a slower shot with the side of the foot to improve accuracy as well as increase the chance that the keeper dives in the wrong direction.

**Figure 5:**
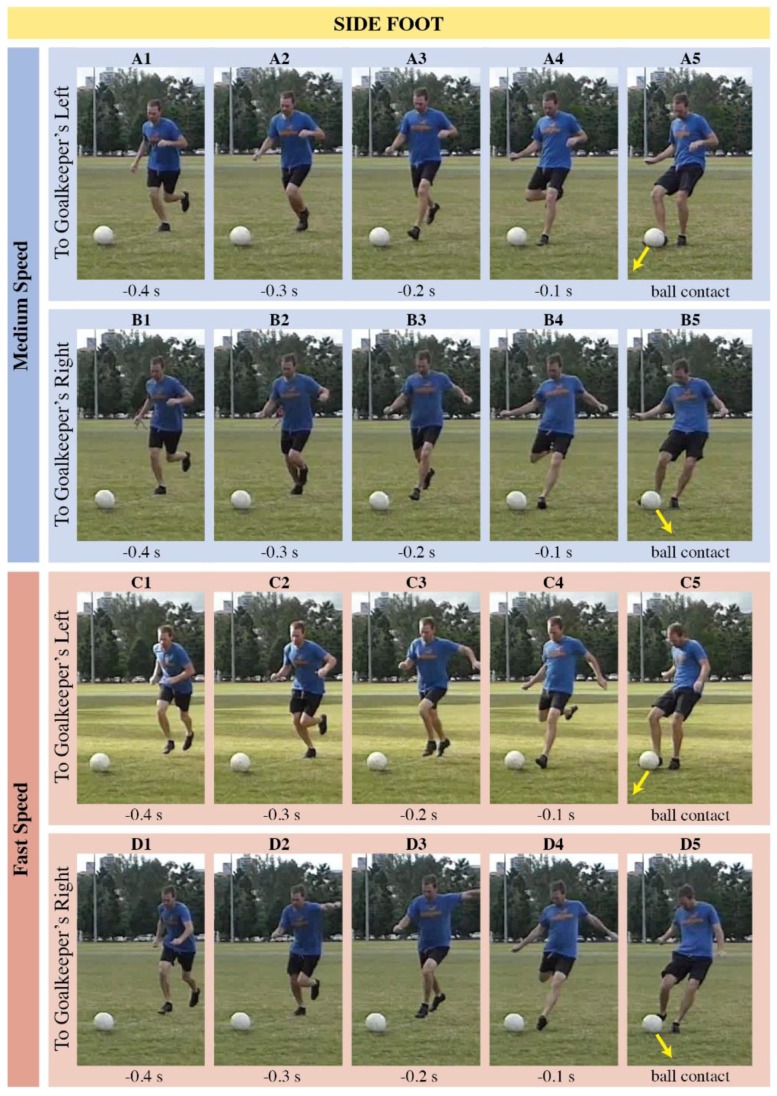
Images of four different shots taken with the side of the foot: medium speed aimed to the reader’s left (panels A1 to A5); medium speed aimed right (panels B1 to B5); fast speed aimed left (panels C1 to C5); and fast speed aimed right (panels D1 to D5). Within each shot, five panels present the final frame of the video participants saw from each of the five occlusion time conditions (−0.4 s, −0.3 s, −0.2 s, −0.1 s, ball contact).

**Figure 6:**
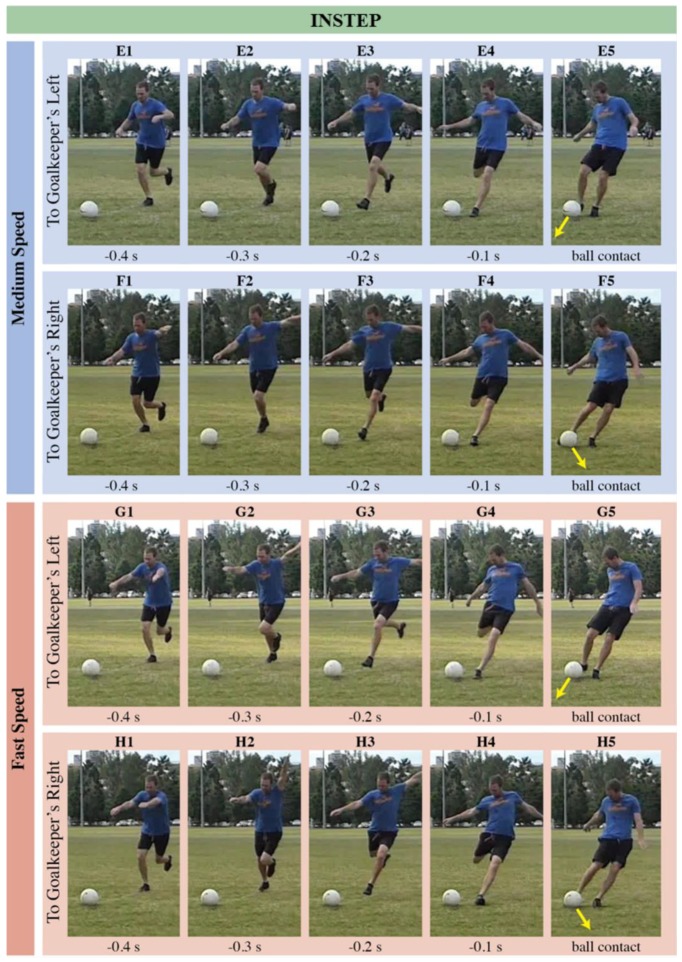
Images of four different shots taken with the instep: medium speed aimed to the reader’s left (panels E1 to E5); medium speed aimed right (panels F1 to F5); fast speed aimed left (panels G1 to G5); and fast speed aimed right (panels H1 to H5). Within each shot, five panels present the final frame of the video participants saw from each of the five occlusion time conditions (−0.4 s, −0.3 s, −0.2 s, −0.1 s, ball contact).

Why is the direction of slower side-foot shots harder for goalkeepers to anticipate? From a goalkeeper’s perspective, movements of the torso, hip, kicking and non-kicking legs, and angle of approach to the ball can all be used to indicate shot direction [7]. Thus, comparing these cues between different types of shots should help us elucidate our results. In Figure 5 and Figure 6, we present time-lapse images of shots with the side of the foot and the instep, respectively. For fast side-foot shots (Figure 5), the kicker orients the left arm, hips, and torso in the direction of the shot early in the kicking action. Differences in the shooter’s posture are obvious −0.3 s before ball contact (compare panels C2 and D2 of Figure 5). Similar cues occur during early stages of shots with the instep, across all speeds (Figure 6). For slower side-foot shots, however, the kicker reveals much less information about the direction of the shot in the earlier stages of kicking (compare panels A2 and B2 of Figure 5). This absence of cues might explain why goalkeepers have more difficulty inferring the direction of slower side-foot shots.

**Figure 7:**
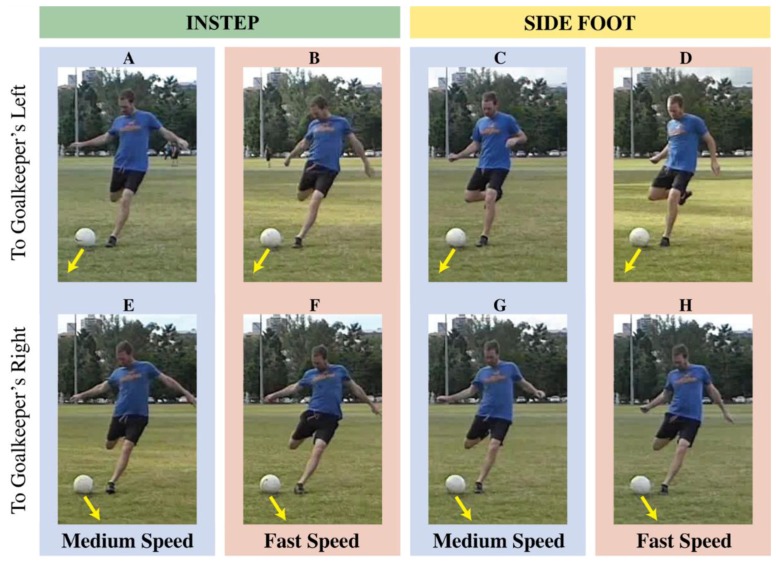
Images of eight shots, one for each combination of kick technique (side-foot {panels C,D,G,H} vs. instep {panels A,B,E,F}), shot speed (medium {panels A,C,E,G} vs. fast {B,D,F,H}), and kick direction (left {panels A,B,C,D} vs. right {E,F,G,H}). All images represent the same point in the shooter’s kicking action, when the non-kicking foot is first planted on the ground.

Across all shot speeds, the direction of instep shots was less predictable than side-foot shots when participants were able to view most of the kicking action up until ball contact. Again, this difference likely relates to the orientation of the body. In Figure 7, we provide images from eight shots of the moment the shooter plants the non-kicking foot (≈−0.1 s before ball contact). At this point, the orientation of the kicker’s hips and torso differ between shots to the left or right, and this difference is exaggerated for fast or side-foot kicks. At any speed, visual cues indicate shot direction more obviously for side-foot shots than instep shots. Furthermore, side-foot shots to the left require greater hip abduction, pointing the knee of the kicking leg toward the direction of the shot. This cue remains absent for instep shots. A goalkeeper could use this cue to predict the direction of a side-foot shot more accurately than the direction of an instep shot. Although our images show only one shooter, the qualitative patterns extend to other shooters in our experiment. A kinematic analysis of multiple shooters would confirm the cues that enable goalkeepers to predict the direction of a shot, and how these are affected by shot speed. The absence of kinematic analysis was a limitation of this study. Regardless, now that we have presented evidence for a trade-off between shot speed and unpredictability, examining the mechanism underlying this relationship should be the focus of future research.

The outcome of a penalty is determined by an interaction between the shooter’s strategy and the goalkeeper’s strategy. For example, shooters can use a “keeper-dependant” strategy, waiting for the goalkeeper to move to a side of the goal before kicking toward the opposite side [25]. Goalkeepers can choose when to dive (or not at all), which is affected by how quickly they can move [27]. In this study, we investigated one aspect of the interaction between shooter and goalkeeper—the relationship between shot speed and unpredictability. While our findings progress the understanding of goalkeeper anticipation in soccer penalties, one must also consider factors such as goalkeeper movement [35], shooting accuracy [30], and shooter deception [7,13] to determine the outcome of a penalty shot.

Our findings have implications across a variety of sports. A similar phenomenon as found here may occur in tennis with the direction of faster shots being easier to predict. While evidence exists that movement amplitude has no influence on predicting shot direction in tennis [15], experiments with human actors (rather than stick figures) are needed to further our understanding of anticipation in tennis. Overarm throwing sports such as baseball or handball could also benefit from replicating our research. Any changes in throwing action between different baseball pitches or intended targets in handball may become more pronounced as throwing speed increases, making their intent easier to read. Athletes in these sports may be less predictable when throwing at sub-maximal speeds. Sports involving evasive manoeuvres such as Rugby League, Rugby Union, Australian Rules Football, and American Football may also be interested in our findings. “Cutting”, where attacking players sharply change running direction, can be a very effective manoeuvre across all football codes, but involves preparatory movements and changes of gait patterns [1,2,36]. There is evidence to suggest that the degree of postural and gait changes required to alter direction is dependent on movement speed [1,37]. When defenders in football games are able to perceive and interpret gait changes in attackers and predict changes in direction [38], they may better anticipate cutting manoeuvres as running speed increases and gait changes become more exaggerated. While the advantage of speed or a deceptive strategy [39] is not to be disregarded across the football codes, there may be situations where attackers benefit from running at sub-maximal speeds to increase both their agility and unpredictability.

Our study is the first to identify a trade-off between the speed of a kick and the predictability of its outcome. In the context of a soccer penalty, we have shown that both the kicker and keeper affect the predictability of a shot. If a keeper is known to dive early, a kicker can maximize unpredictability with a slow side-foot shot. However, if a keeper tends to dive late, a kicker must use the instep to maximize unpredictability, which necessarily reduces accuracy. Thus, the optimal strategy depends on the keeper’s behaviour and the relative benefits of speed, accuracy, and unpredictability within each situation. A game theoretical perspective is needed to understand how these trade-offs determine the best strategies of each player.

## Figures and Tables

**Table 1:** Based on Akaike information criterion (AIC), we ranked statistical models of the probability of predicting the correct direction of a kick (left vs. right). Only models with a likelihood of >0.001 are listed below. For each model, we report the difference between its AIC and the AIC of the most likely model (ΔAIC) and the likelihood that the model describes the data better than other models (*w*).

Model	Degrees of Freedom	AIC	∆AIC	*w*
(1) speed + technique + occlusion + (speed × technique) + (technique × occlusion)	14	−18615.58	0	0.78
(2) speed + technique + occlusion + (speed × occlusion) + (speed × technique) + (technique × occlusion)	22	−18608.88	2.62	0.21

**Table 2:** Parameters of the most likely model of the probability of predicting the correct direction of a kick (left vs. right).

Parameter	Estimate	Standard Error	z	*p*
intercept	1.350	0.068	19.91	<0.001
medium speed	−0.057	0.068	0.83	0.41
low speed	0.091	0.102	0.89	0.37
side kick	0.821	0.081	10.17	<0.001
−0.1 s occlusion	−0.445	0.083	5.39	<0.001
−0.2 s occlusion	−0.470	0.084	5.60	<0.001
−0.3 s occlusion	−0.678	0.093	7.28	<0.001
−0.4 s occlusion	−0.809	0.069	11.75	<0.001
medium speed side kick	−0.151	0.063	2.40	0.02
low speed side kick	−0.297	0.067	4.45	<0.001
side kick −0.1 s occlusion	−0.252	0.093	2.69	0.01
side kick −0.2 s occlusion	−0.870	0.090	9.62	<0.001
side kick −0.3 s occlusion	−0.816	0.089	9.17	<0.001
side kick −0.4 s occlusion	−0.919	0.088	10.41	<0.001
medium speed −0.1 s occlusion	−0.029	0.076	0.38	0.70
low speed −0.1 s occlusion	−0.051	0.110	0.46	0.65
medium speed −0.2 s occlusion	−0.036	0.085	0.42	0.67
low speed −0.2 s occlusion	−0.048	0.105	0.45	0.65
medium speed −0.3 s occlusion	−0.035	0.083	0.42	0.67
low speed −0.3 s occlusion	−0.073	0.147	0.49	0.62
medium speed −0.4 s occlusion	−0.003	0.049	0.05	0.96
low speed −0.4 s occlusion	−0.040	0.091	0.44	0.66
medium speed side kick −0.1 s occlusion	−0.001	0.023	0.05	0.96
low speed side kick −0.1 s occlusion	−0.002	0.029	0.06	0.95
medium speed side kick −0.2 s occlusion	0.000	0.016	0.02	0.98
low speed side kick −0.2 s occlusion	−0.002	0.035	0.06	0.95
medium speed side kick −0.3 s occlusion	0.000	0.015	0.02	0.98
low speed side kick −0.3 s occlusion	−0.001	0.024	0.05	0.96
medium speed side kick −0.4 s occlusion	0.000	0.015	0.01	0.99
low speed side kick −0.4 s occlusion	−0.001	0.026	0.06	0.96

**Figure A1:**
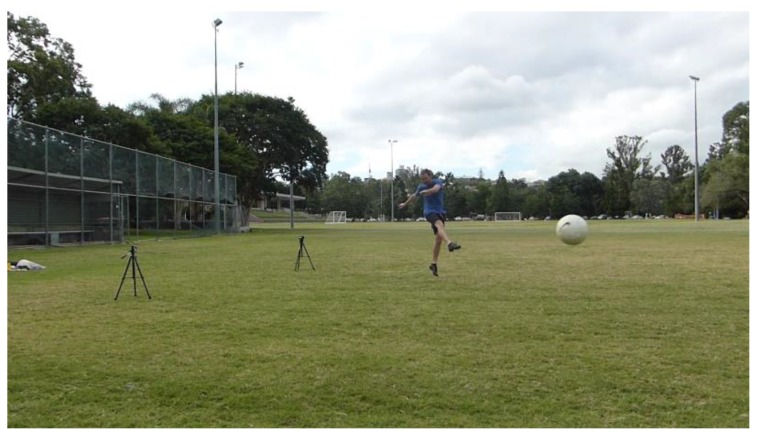
Example of a shot to the right.

## Supplementary Materials

The following are available online at http://www.mdpi.com/2075-4663/6/3/73/s1. Figure S1: Model results for goalkeepers only; Video S0: Ball contact; Video S1: −0.1 s; Video S2: −0.2 s; Video S3: −0.3 s; Video S4: −0.4 s.

## Appendix A

*Survey Instructions*

Hi,

Thanks for taking part in this experiment. It should only take 10–15 min of your time.

You are about to find out how good a soccer goalkeeper you are. You will watch 60 different videos of soccer players shooting a penalty at you and your task is to guess if their shot went to **your** left or right. Each video will stop before the ball is actually kicked, so you have to make your prediction based on their body cues. Some videos will stop right at ball contact, while others stop at various points during the players run-up/kicking action, so the amount of information you have to make your decision will vary.

You will only be able to watch each video once before making your prediction. Try not to think too much about it, just go with your gut instinct. You should be making your prediction within a couple of seconds of each video finishing.

You will have 10 practice trials to get familiar with how it all works, and you will find out if your predictions were correct during the practice trials. When you move onto the 60 test trials, you will not get any feedback on whether your predictions were correct. No kicks you watch throughout this survey will be repeated.

To make your prediction click on either the “left” or “right” button. You will see these to either side below the video.

In the example below (Figure A1), this shot has gone to the right.

Please note:

Participation in this study is voluntary. You do not have to take part in this project and you are free to withdraw at any time. Your withdrawal would not be held against you in any way. You may not directly benefit from participation in this study. For analysis, electronic information will be de-identified with ID numbers, so no individual will be personally identifiable. All information will be stored on a password protected computer at the University of Queensland. All reports generated from this research will present data either in aggregated form or in a de-identified manner.

This study has been reviewed and approved by one of the Human Research Ethics Committees at The University of Queensland. If you have any questions about this research study or your participation, please contact: Andrew Hunter at a.hunter@uq.edu.au

Should you wish to discuss the study with somebody who is someone not directly involved, you can contact the Ethics Officer on (07) 3365 3924 or humanethics@research.uq.edu.au.

If you’re happy to participate, hit the ‘>>’ arrow at the bottom right to continue.

*Demographic Questions*

What best describes your soccer playing experience under the age of 18?

○ Never played
○ I occasionally kicked a ball around with friends and/or played in social competitions
○ I regularly played in organised leagues—small-sided or 11-a-side

Was your experience under 18 primarily as an out-field player or goalkeeper?

○ Out-field
○ Goalkeeper

What best describes your soccer playing experience over the age of 18?

○ Never played
○ I have occasionally kicked a ball around with friends and/or played in social competitions
○ I have regularly played in organised leagues—small-sided or 11-a-side
○ I have regularly played in semi-professional leagues—some players are payed to play
○ I have regularly played in fully professional leagues—all players are paid, and this is their main source of income

Was your experience over 18 primarily as an out-field player or goalkeeper?

○ Out-field
○ Goalkeeper

What is your age in years?

What is your gender?

○ Male
○ Female
○ Other

**Table A1:** Tukey HSD comparisons identifying differences in correctly guessing shot direction based on soccer playing experience over the age of 18. (1) never played, (2) played socially, (3) amateur player, (4) semi-professional.

Comparison	∆ Mean	Lower 95% CI	Upper 95% CI	*p*
2-1	0.064	0.040	0.089	<0.001
3-1	0.074	0.049	0.099	<0.001
4-1	0.079	0.049	0.109	<0.001
3-2	0.010	−0.013	0.033	0.678
4-2	0.015	−0.014	0.043	0.555
4-3	0.005	−0.024	0.033	0.975
