# Supplementary material for: Anticipating the Direction of Soccer Penalty Shots Depends on the Speed and Technique of the Kick
Source: Sports (Basel). 2018 Jul 29;6(3):73. doi: 10.3390/sports6030073 (PMC6162804; doi:10.3390/sports6030073)
Supplement: Supplementary file 1 [file sports-06-00073-s001.zip › Sports-330385-SI-revise.docx]

Supplementary

Anticipating the Direction of Soccer Penalty Shots Depends on the Speed and Technique of the Kick


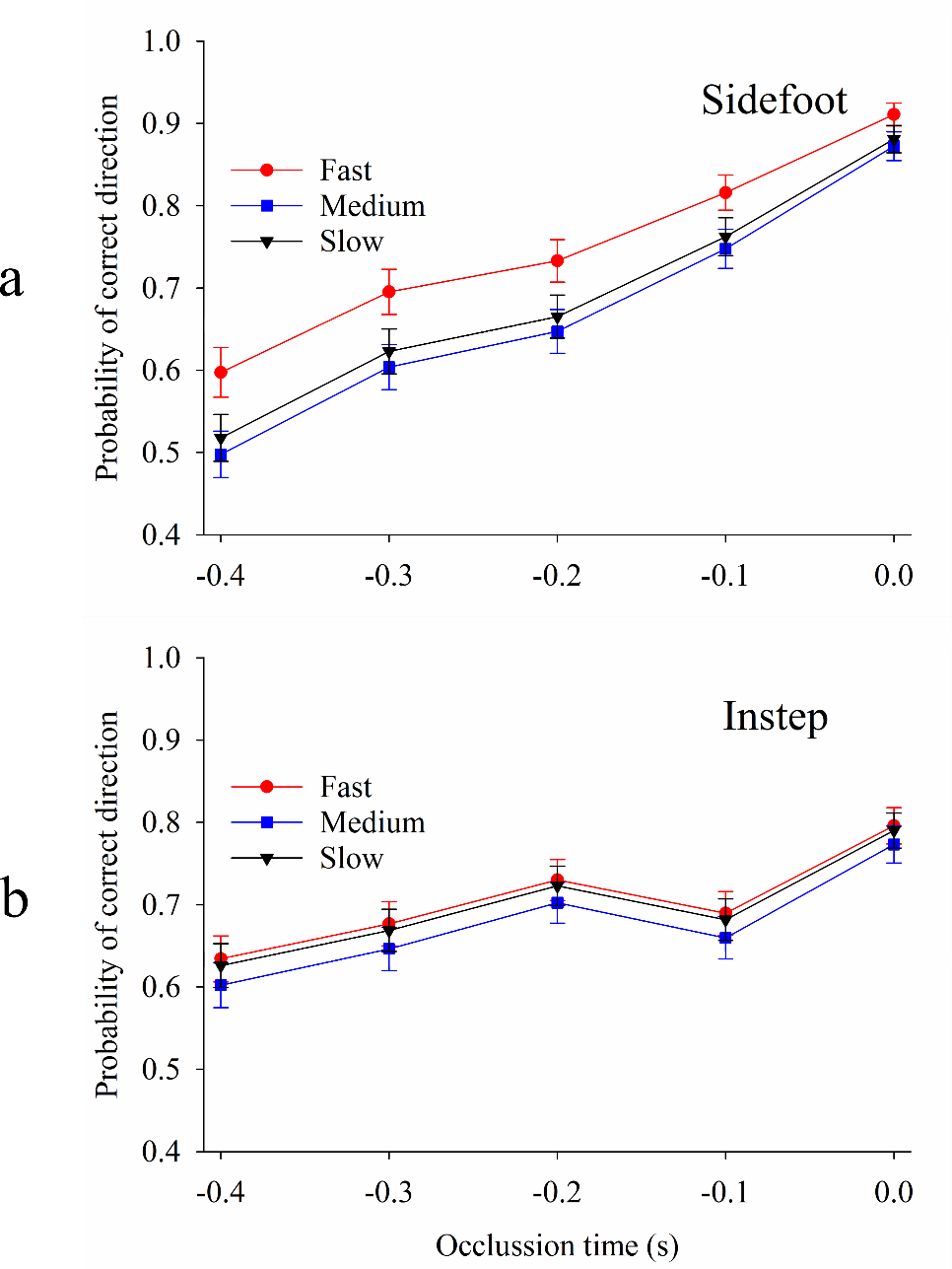


**Figure S1.** For participants over the age of 18 with goalkeeping experience, probability of correctly guessing shot direction dependent on occlusion time and shot speed. Probabilities and Standard Error bars calculated using averaged parameter estimates from statistical model. (**a**) Side-foot shots. (**b**) Instep shots.
