# Supplementary figures and images for: Anticipating the Direction of Soccer Penalty Shots Depends on the Speed and Technique of the Kick
Source: Sports (Basel). 2018 Jul 29;6(3):73. doi: 10.3390/sports6030073 (PMC6162804; doi:10.3390/sports6030073)

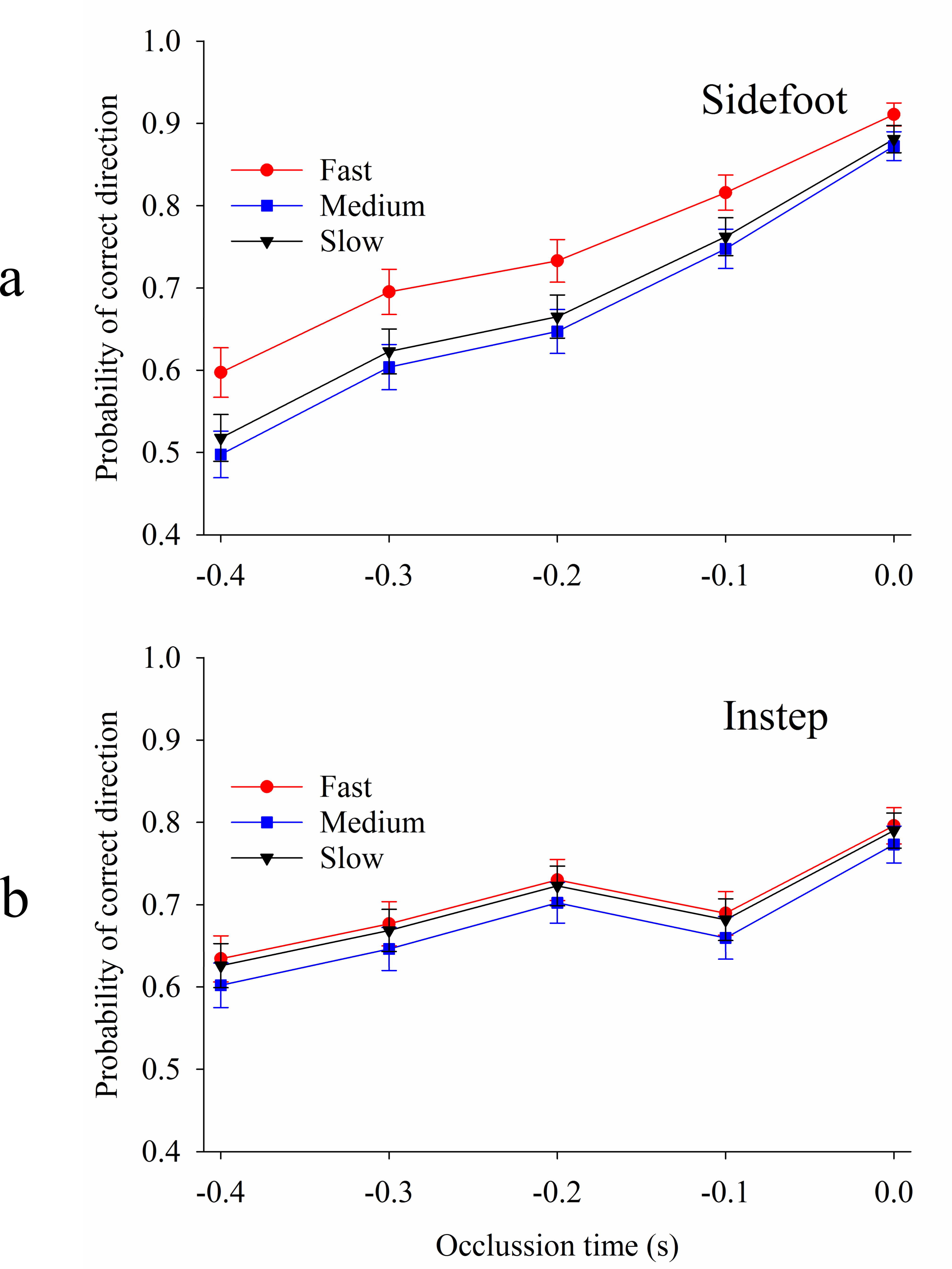

Supplement: Supplementary file 1 [file sports-06-00073-s001.zip › Hunter et al - video - Sports - Supplementary Figure 1.png]
